# Effective hemodynamic monitoring

**DOI:** 10.1186/s13054-022-04173-z

**Published:** 2022-09-28

**Authors:** Michael R. Pinsky, Maurizio Cecconi, Michelle S. Chew, Daniel De Backer, Ivor Douglas, Mark Edwards, Olfa Hamzaoui, Glenn Hernandez, Greg Martin, Xavier Monnet, Bernd Saugel, Thomas W. L. Scheeren, Jean-Louis Teboul, Jean-Louis Vincent

**Affiliations:** 1 Critical Care Medicine, Bioengineering, Cardiovascular Diseases, Anesthesiology and Clinical & Translations Medicine, University of Pittsburgh, Pittsburgh, PA USA; 2 Anesthesiology and Intensive Care, Department of Biomedical Sciences, MEDTEC School, Humanitas University Pieve Emanuele, Milan, Italy; 3 Anaesthesia and Intensive Care Medicine, IRCCS Humanitas Research Hospital, Rozzano, Milan, Italy; 4 Anesthesiology, Intensive Care and Acute Medicine, Linkoping University Hospital, Linkoping, Sweden; 5 Intensive Care Department, CHIREC Hospitals, Intensive Care, Universite Libre de Bruxelles, Brussels, Belgium; 6 Medicine, Pulmonary Sciences & Critical Care Medicine, University of Colorado Medical School and Denver Health Medical Center, Aurora, CO USA; 7 Anaesthesia & Perioperative Medicine, University Hospital Southampton, University of Southampton, Southampton, UK; 8 Medical Intensive Care, Robert Debré Hospital, University Hospitals of Reims, Reims, France; 9 Departamento de Medicina Intensiva, Facultad de Medicina, Pontificia Universidad Católica de Chile, Santiago, Chile; 10 Medicine, Division of Pulmonary, Allergy, Critical Care and Sleep Medicine, Emory University & Grady Memorial Hospital, Atlanta, GA USA; 11 Intensive Care, Paris-Saclay University Hospitals, Paris-Saclay University, Paris, France; 12 Anesthesiology, Department of Anesthesiology, Center of Anesthesiology and Intensive Care Medicine, University Medical Center Hamburg-Eppendorf, Hamburg, Germany; 13 Anesthesiology, University Medical Center Groningen, Groningen, Netherlands; 14 Therapeutics and Intensive Care Medicine, Paris-Saclay University Hospitals, Paris-Saclay University, Paris, France; 15 Department of Intensive Care Medicine, Erasme University Hospital, Université Libre de Bruxelles, Brussels, Belgium

## Abstract

Hemodynamic monitoring is the centerpiece of patient monitoring in acute care settings. Its effectiveness in terms of improved patient outcomes is difficult to quantify. This review focused on effectiveness of monitoring-linked resuscitation strategies from: (1) process-specific monitoring that allows for non-specific prevention of new onset cardiovascular insufficiency (CVI) in perioperative care. Such goal-directed therapy is associated with decreased perioperative complications and length of stay in high-risk surgery patients. (2) Patient-specific personalized resuscitation approaches for CVI. These approaches including dynamic measures to define volume responsiveness and vasomotor tone, limiting less fluid administration and vasopressor duration, reduced length of care. (3) Hemodynamic monitoring to predict future CVI using machine learning approaches. These approaches presently focus on predicting hypotension. Future clinical trials assessing hemodynamic monitoring need to focus on process-specific monitoring based on modifying therapeutic interventions known to improve patient-centered outcomes.

## Philosophy of hemodynamic monitoring

Hemodynamic monitoring techniques can identify cardiovascular insufficiency (CVI) and guide personalized hemodynamic therapies when linked to clinical examination to assess perfusion adequacy. Effective hemodynamic monitoring to achieve these goals should be associated with improved outcomes. Still, no hemodynamic monitoring device will improve outcomes unless coupled to an appropriate and effective treatment [1]. Clinical data suggest that excessive fluid resuscitation worsens outcomes [2, 3]. Using dynamic variables of fluid responsiveness during resuscitation limits fluid infusion in non-responsive patients [4, 5]. Similarly, inotropic agents can be given to achieve the maximum benefit at the least possible dose when titrated to hemodynamic targets [6]. Hemodynamic monitoring can be invasive or non-invasive [7]. Increasingly invasive monitoring usually supplies more stable and pluripotential display that may potentially allow for better titration of care. Debate continues as to the degree of invasiveness and monitoring frequency needed to define benefit. Highly granular data collection is expensive and subject to increased artifacts.

## The problem

It is difficult to define monitoring effectiveness. To monitor or not and to treat or not both have outcomes which can be good or bad. Improved outcomes are directly related to a more effective use of therapies while minimizing iatrogenic effects by limiting those therapies in patients less likely to benefit from their use. Such analyses will probably be patient, process and condition specific. We will focus on effectiveness of monitoring-linked resuscitation strategies by three lenses (Table 1). First, process-specific monitoring, that by its structure allows for non-specific identification of new onset CVI in at-risk patients. Second, defining patient-specific cardiovascular states to personalize and optimize resuscitation approaches. And third, hemodynamic monitoring to identify clinically relevant decompensation earlier. Relevant clinical outcomes need to be patient-centric: decreased ICU and hospital length of stay, shorter time on mechanical ventilation, time to tolerating oral intake, reduced incidences of acute kidney injury (AKI) and other acute illness complications.

**Table 1:** Outcome effectiveness targets for hemodynamic monitoring-guided acute care*

Setting	Monitor-treatment	Outcome
Perioperative	Pre-optimization (CO)	Reduced complications
		Reduced ventilator time
		Reduced ICU/hospital LOS
	Functional hemodynamic monitoring	Decreased infused volume
		Decreased lac-time
	Hypotension prediction	Decreased hypotension time
Emergency Department	Sepsis resuscitation SSG	Decreased mortality
	Functional hemodynamic monitoring sepsis	Decreased infused volume
		Lower lac-time
		Decrease hypotension time
ICU resuscitation	Functional hemodynamic monitoring sepsis	Decreased infused volume
		Decreased hypotension time
ICU management	Stabilization/de-escalation (Eadyn)	Rapid norepinephrine weaning

*CO cardiac output, *Eadyn* dynamic arterial elastance, *ICU* intensive care unit, *LOS* length of stay, *lac-time* duration of time serum lactate is > 2.0 mmol/l, *SSG* surviving sepsis guidelines

### Process-specific monitoring

Electrocardiographic (ECG) monitoring in patients with acute coronary syndrome often identifies clinically relevant arrhythmias that indicate worsening ischemia and/or progression to malignant arrhythmias before death [8]. Since there are effective treatments for many causes of cardiac arrhythmias and ST segment changes, ECG monitoring is generally used and presumed to be effective in those patients. However, ECG monitoring in non-acute coronary syndrome populations is poorly validated [8]. Similarly, monitoring of arterial oxygenation using pulse oximetry derived pulse oxygen saturation (SpO_2_) is universally used in emergency transport, high dependency units and intra-operatively. Although useful in identifying supplemental oxygen resistant hypoxemia, a large scale randomized clinical trial demonstrated no measurable benefit in perioperative patients [9]. Accordingly, even though the information provided may be interesting in broader populations, the effectiveness is often restricted in selected syndromes.

#### Advanced hemodynamic monitoring for surgical patients

Since the effects of intra-operative CVI are devastating and because both anesthesia and surgical manipulations alter cardiovascular function close monitoring of blood pressure (BP), heart rate, and SpO_2_ is mandatory during surgery without validation [10]. Advanced hemodynamic monitoring such as pulmonary artery catheterization [11] (e.g., cardiac output (CO) and dynamic cardiac preload variables) is usually only performed in patients having major surgery and in high-risk patients. They are not routinely used outside of cardiac surgery, organ transplant or major abdominal surgery. Invasive arterial pulse wave analysis [12] and transesophageal Doppler [13] can also estimate CO and volume responsiveness. The clinical applicability of non-invasive methods like finger-cuff, pulse wave transit time and bioimpedance/bioreactance [14] is still being analyzed. Though intraoperative hypotension and postoperative AKI and myocardial injury are tightly coupled, it is unknown if this association is *causal* [15]. In a sub-study of the POISE-II trial, hypotension occurring up to 4 days postoperatively was associated with 30-day myocardial infarction and death [16]. While universally targeting higher intraoperative mean arterial pressure (MAP)(≥ 75 vs. ≥ 60 mmHg) does not reduce postoperative complications in patients undergoing elective major non-cardiac surgery [17], individualizing intraoperative MAP targets based on preoperative values reduced postoperative systemic inflammation and organ failure in major non-cardiac surgery patients [18]. Using advanced perioperative monitoring without accompanying treatment algorithms did not improve any outcome [19].

Hemodynamic monitoring-based preoptimization goal-directed therapy (GDT) algorithms (preoptimization) aim to improve global oxygen delivery (DO_2_) by targeting hemodynamic endpoints using fluids, inotropes, vasopressors and red blood cells [20]. Initial meta-analyses of small trials using preoptimization algorithms were inconclusive [21, 22]. The OPTIMISE trial targeted CO optimization in major noncardiac surgery. It did not demonstrate a significant reduction of complications and mortality (absolute risk reduction, 6.8%, 95% CI, − 0.3 to 13.9%; *P* = 0.07) [23], adding these results in an updated meta-analysis showed significant reduction in complications (RR 0.77 [95% CI, 0.71–0.83%). The subsequent FEDORA trial enrolled low-moderate risk patients having elective, major noncardiac surgery, randomizing patients to a GDT algorithm targeting MAP and CO, finding a significant reduction in postoperative complications [10], including the incidence of AKI, acute respiratory distress syndrome (ARDS), pneumonia and non-cardiogenic pulmonary edema. The benefits of GDT in both trials were also driven by a reduction in postoperative infections, now the primary outcome in the ongoing OPTIMISE-II trial (ISRCTN39653756) [24]. While OPTIMISE used a CO-driven management algorithm, FEDORA used a combined MAP-CO approach. Finally, a small single-center trial suggested that personalized CO-guided management maintaining baseline CO reduced postoperative complications in elective major abdominal surgery patients [25]. However, in a trial of 482 high-risk elective abdominal surgery patients such perioperative GDT did not improve outcomes [26], nor did monitoring continued following surgery for 24 h. A recent study comparing CO versus MAP guided fluid therapy in bowel obstruction or gastrointestinal perforation surgery found no outcome difference [27]. Thus, continuing monitoring and optimization following surgery may not be beneficial [26]. A subsequent larger trail is ongoing (FLO-ELA, ISRCTN14729158).

### Individualizing the assessment of cardiovascular reserve

Although every patient is different, the practical translation of that into patient-specific treatment algorithms has been slow. Furthermore, when hemodynamic monitoring is used to assess therapeutic challenges in an unstructured fashion, the FENICE study revealed profound practice variability [28]. The reference for fluid resuscitation is the fluid challenge which evaluates the patient response to a fluid bolus given over a short period of time [29]. The fluid administration should be stopped if the CO response is negligible.

### Defining circulatory sufficiency

Although it is relatively easy to identify variables to trigger resuscitation, it is less clear on when to stop it. Effective resuscitation is usually measured by return of normal end-organ function. However, such clinically relevant end-points usually occur long after sufficiency is achieved. Therefore, clinicians often resuscitative beyond levels needed to attain sufficiency, leading to volume overload and excess vasoactive drug exposure. Mixed venous oxygen saturation (SvO_2_) and arterio-venous O_2_ and CO_2_ (v-aCO_2_) gradients help to identify tissue hypoperfusion. Central venous oxygen saturation (ScvO_2_) and v-aCO_2_ gap can be used as a surrogate for SvO_2_ [30]. SvO_2_ < 70% documents circulatory stress while v-aPCO_2_ > 6 mmHg is consistent with tissue hypoperfusion [31]. However, these measures require invasive monitoring and not routinely used to guide resuscitation in clinical trials [32]. Measures of forearm tissue O_2_ saturation (StO_2_) by near infrared spectroscopy in response to a transient vascular occlusion test identifies occult circulatory shock, is non-invasive and easy to perform [33], while steady state StO_2_ is minimally informative [34]. Hyperlactatemia is considered a marker tissue hypoxia [35]. Targeting lactate reductions in patients with shock of different etiologies was associated with less organ dysfunction, mechanical ventilation and ICU length of stay and when adjusted for predefined risk factors decreased mortality [36]. However, persistent hyperlactatemia has many causes, and appropriate lactate decreases to resuscitation are slow. Pursuing lactate normalization increases the risk of fluid overload, especially when other tissue hypoperfusion indices are absent [35, 37], as suggested by a post hoc analysis of a recent major trial [38]. The use of lactate as a resuscitation target requires significant clinical interpretation [37].

Capillary refill time (CRT), a costless universally available technique with unique characteristics that may be pivotal for assessing circulatory effectiveness and is more sensitive than skin mottling to identify CVI [39]. Measuring CRT is easily taught and shows good consistency across observers if performed in a standardized fashion [40]. CRT also exhibits a fast kinetics of recovery after septic shock resuscitation and may be considered a flow-sensitive variable to evaluate response to fluid boluses or vasoactive titration [32, 39]. Its rapid normalization is associated with higher survival and may reflect an earlier CVI stage with preserved hemodynamic coherence between macro and microcirculations [31, 41–43]. Targeting a normal CRT was associated with less treatment intensity, organ dysfunction and a trend to lower mortality as compared to targeting normalization of lactate in early septic shock [41, 42]. Presently, it seems reasonable to define circulatory sufficiency as a state when most of the above variables reach target values [37].

### Predicting volume responsiveness

Fluid administration is the first treatment undertaken in most patients with CVI. However, volume expansion poses two problems [44]. CO increases in only half of the CVI patients, because of the inconsistent relationship between stroke volume and cardiac preload. Whereas a positive fluid balance worsens the patients’ outcome [44, 45]. Thus, fluids are treatments with inconsistent efficacy, significant deleterious effects and a significant risk of overdose [44]. Resuscitation strategies that target static hemodynamic endpoints alone, such as MAP and central venous pressure, also result in outcomes no different than non-protocolized care. This arises because static measures cannot predict fluid responsiveness, thus those patients are still given fluids causing a positive fluid balance [46]. Fluids responsiveness should be identified before starting volume expansion to treat CVI. Several hemodynamic tests and indices accurately predict volume responsiveness and have been extensively reviewed elsewhere [47]. Respiratory variation of arterial pulse pressure (PPV) and stroke volume (SVV) to positive-pressure ventilation, tidal volume challenge and end-expiratory occlusion tests are based on heart–lung interactions. Others, such as the passive leg raising test and the mini fluid challenge, mimic the effects of a standard fluid bolus [48, 49]. Their use to guide resuscitation efforts have been widely endorsed.

A meta-analysis of 14 randomized control post-surgical trials before 2014 (961 participants) showed that a dynamic assessment of volume responsiveness GDT reduced post-operative morbidity (odds ratio 0.51, 95%CI 0.34 to 0.75%; *P* < 0.001), related to decreased cardiovascular, infectious and abdominal complications. The ICU length of stay was reduced (− 0.75 days, 95% CI − 1.37 to − 0.12; *P* = 0.02) [50]. Two subsequent meta-analyses found similar results [51, 52]. One surgical ICU meta-analysis (11 studies, 1015 patients), using PPV or SVV was associated with decreased mortality (odds ratio 0.55, 95% CI 0.30 to 1.03) and ICU-related costs per patient (− 1619 US$, 95% CI − 2174 to − 1063 US$) [52].

Four randomized trials in patients with early septic shock were done assessing volume responsiveness as a positive response to passive leg raising [53–56]. The primary goal, a reduction in fluid balance, was universally seen. In the largest study, the need for renal replacement therapy was reduced [54]. A reduction in mortality, not the primary goal of these studies, was not observed, nor confirmed by two meta-analyses [57, 58]. In an observational study in septic shock patients, the use of volume responsiveness test was associated with a better prognosis [59]. Targeting therapy by the documenting volume responsiveness did not delay fluid administration. Applying volume responsiveness limits to fluid administration in CVI treatment avoided administering fluids to patients who will not benefit from it, and it does not induce any deleterious effect [60]. Although no differences in mortality were observed, mortality may not be the ideal objective in critically ill patients [61].

### Minimizing the volume of resuscitation using vasopressors early

At the early phases of resuscitation, fluid and vasopressor administration is often required [56]. While the classical approach suggested to initiate vasopressors after fluid completion in persistently hypotensive patients, initiating early vasopressors limits the administered fluid volume and minimizes hypotension time. In experimental septic shock, early administration of norepinephrine reduced lactate and decreased the volume of fluids required to achieve hemodynamic resuscitation [62]. In humans with septic shock, observational data suggest that delayed vasopressor administration is associated with increased mortality [63–65]. Early vasopressor use is physiologically sound in vasodilatory shock, as it reverses the vasodilation-induced shift of blood from the stressed to unstressed volume. Using a propensity matched analysis of sepsis patients [66], early introduction of vasopressors was associated with less fluid and improved survival, though not confirmed in another retrospective study which found that similar amounts of fluids were given in early and later vasopressor groups and the use of vasopressors early was associated with a higher mortality [67]. Whether this association reflects more severely ill patients requiring vasopressors early or the independent impact of vasopressors is unknown. In a pilot randomized trial of sepsis with hypotension, administration of fixed dose norepinephrine (0.05 μg kg^−1^ min^−1^) within 1 h of hypotension resulted in less fluid administration, less cardiogenic pulmonary edema, fewer arrhythmias and a lower mortality [68]. The CLOVERS trial comparing crystalloid liberal or vasopressors in the early resuscitation in sepsis from the PETAL network [46] was stopped for futility, while the CLASSIC trail showed no difference in mortality between these two treatments [69].

### Assessing arterial tone and predicting arterial pressure responses to hypotension and vasopressor weaning

Pathological decreased vasomotor tone (vasoplegia) is a common cause of hypotension. Vasoplegia will limit the BP increase to fluids even if volume responsive. Low diastolic pressure may indicate vasodilation and need of vasopressors. The diastolic shock index (heart rate/diastolic blood pressure) > 2.5 was associated with increased risk of death [70]. As proposed by Pinsky in 2002, the PPV to SVV ratio, termed dynamic arterial elastance (Ea_dyn_), estimates the dynamic changes in pressure as flow is varied in hypotensive patients [1]. However, in normotensive patients Ea_dyn_ will vary inversely with flow to maintain a constant blood pressure through baroreceptor feedback. Thus, Ea_dyn_ is less useful in normotensive patients [71]. Also, Ea_dyn_ is not arterial elastance, though it does reflect ventriculo-arterial coupling [72]. Several studies in both mechanically ventilated and spontaneously breathing patients documented Ea_dyn_ < 1.0 in a hypotensive volume responsive patient predicts whose blood pressure will not increase with increasing CO [6, 73–75]. No studies have used Ea_dyn_ to initiate vasopressor therapy and assess its impact of organ perfusion and outcomes.

Ea_dyn_ can be used to wean vasopressors in normotensive vasopressor-dependent patients. Guinot et al. [76] in septic shock patients and Vos et al. [77] in the perioperative setting showed that Ea_dyn_ identified those norepinephrine-dependent patients whose with a Ea_dyn_ > 1 could have their vasopressor decreased without hypotension, whereas if Ea_dyn_ was < 1 vasopressor weaning as associated with hypotension. Using Ea_dyn_ thresholds to start norepinephrine weaning in post-cardiac surgery vasoplegia patients Guinot et al. showed a 50% reduction in both norepinephrine time and dose, less arrhythmias and a one-day decrease in ICU length of stay [78].

### When less treatment is more

Less aggressive treatment is sometimes associated with better outcomes in critically ill patients. Less red blood cell transfusions, conservative fluid management and lower tidal volume mechanical ventilation in ARDS patients improve outcomes. Similarly, the non-specific use pulmonary artery catheterization may have led to greater resuscitation and worse survival [79]. Similarly, a too restrictive approach may also be detrimental as both approaches may have adverse results if used systematically, effective hemodynamic monitoring may help to achieve the optimal therapy at the individual level. Dynamic hemodynamic monitoring approaches predict fluid responsiveness mitigating the risks of excessive fluid administration identifying those who benefit from fluids [3]. Similarly, when the decision to start an inotrope has been made based on the clinical picture, together with the analysis of perfusion indices and the confirmation of abnormal cardiac function by bedside echocardiography, inotropes can be titrated to achieve the maximum benefit for the least possible dose because high dosages and sustained use can be toxic [80]. Hemodynamic monitoring-derived dynamic measures guiding therapy in septic shock patients leads to less fluid administration with similar or superior outcomes [54].

### Optimizing cardiovascular support in circulatory shock: salvage, optimization, stabilization, de-escalation

No clinical trials have compared hemodynamic monitoring-guided resuscitation from shock to no hemodynamic monitoring conditions, thus most studies compare threshold values of monitor-specific hemodynamic values on specific outcomes when specific treatments or drugs are used to reach those hemodynamic values. The monitoring and management of CVI can be separated into “phases” of care, defined by changing monitoring and management priorities, defined as salvage, optimization, stabilization and de-escalation [81]. During the salvage phase, it is essential to correct profound hypotension as it is a strong predictor of mortality [82] and to identify and treat severe cardiac dysfunction leading to a low CO. During this phase therapies restore and/or maintain MAP > 65 mmHg [82] or more in previously hypertensive patients [83]. Thus, invasive arterial pressure monitoring to guide therapy is indicated if initial fluid bolus does not restore MAP. Echocardiography should be performed as soon as possible to check cardiac function [84, 85]. The level of invasiveness is the subject of other expert consensus statements.

During the optimization phase, the main goal is to adapt DO_2_ to cellular oxygen demand. At the macro-circulatory level, inadequate DO_2_ can be due to hypoxemia, low hemoglobin concentration, and/or inadequate CO. All cause a low SvO_2_ [86]. The specific etiology should be sought using hemodynamic monitoring and resolved by appropriate therapies. At that stage in resuscitation, dynamic tests of fluid responsiveness [47] and echocardiography are useful to guide fluid and vasoactive drug therapy, limiting fluid resuscitation in non-fluid responsive patients. In some forms of distributive shock, ScvO_2_ can be > 70% despite ongoing CVI due to impairment of oxygen extraction [84, 86]. A v-aPCO_2_ > 6 mmHg (or > 0.8 kPa) identifies patients for whom an increase in CO may be beneficial in sustaining organ perfusion despite a SvO_2_ > 70%. If the v-aPCO_2_ is < 6 mmHg (or < 0.8 kPa), it is unlikely that increasing CO would reverse organ hypoperfusion. In those situations where despite initial resuscitation, persistent organ dysfunction and evidence of tissue hypoperfusion persists (e.g., hyperlactatemia, metabolic acidosis, delayed CRT) despite a normal (or high) ScvO_2_ and normal v-aPCO_2_, marked microcirculatory disorders and/or mitochondrial dysfunction, poorly responsive to macrohemodynamic therapeutic manipulations, probably exist. This situation can occur during septic shock and is termed refractory shock. Response to resuscitative measures can be assessed by noting the trends in blood lactate [36] or CRT [32, 87]. Expert consensus suggests that shock unresponsive to initial hemodynamic therapy based on clinical assessment, echocardiography, lactate, CRT and variables measured from blood samples need hemodynamic monitoring escalated to transpulmonary thermodilution (TPTD) and pulmonary artery catheter (PAC) systems to define better the limits of resuscitation versus harm [84]. No clinical trials have assessed this monitoring escalation approach.

With successful treatment, stabilization should follow the optimization. This phase is characterized by both the absence of shock, and lack of imminent threat of shock. Echocardiography may help to wean inotropes when cardiac dysfunction is resolving. Fluid removal is often needed as vascular unstressed volume reverts to baseline levels and third space fluids are resorbed [88]. At this stage, if fluid unresponsiveness is detected, fluid removal should not cause CO reductions [47]. Reappearance of hypoperfusion markers during diuresis may indicate that the rate of fluid removal should be limited or stopped.

### Optimizing cardiovascular support of CVI during acute lung injury

ARDS patients can suffer CVI due to associated sepsis. ARDS is often associated with right ventricular dysfunction and increase pulmonary capillary permeability. Thus, potential major detrimental consequences of fluid administration on hemodynamics may occur. The causes of ARDS can be complex and causes of death are multiple, making it difficult to demonstrate any benefit on survival from hemodynamic therapeutic protocols. Since no monitoring device has been demonstrated to cause harm per se, it seems unreasonable to manage such complex patients without appropriate invasive hemodynamic tools since clinical and biochemical signs are often misleading [80, 84]. Bedside echocardiographic evaluation is necessary to diagnose and direct the management of these patients in both a static and dynamic fashion but is not well suited to continual monitoring.

TPTD reports extravascular lung water (EVLW), a measure of the amount of pulmonary edema, and pulmonary vascular permeability index (PVPI), a measure of the lung capillary leak. Both variables can be viewed as markers of lung tolerance to fluid administration. Since TPTD is coupled with the pulse contour analysis technique in the same device, such systems enable assessment of volume responsiveness. These systems provide assessment of the benefit/risk balance of fluid infusion: volume responsiveness to assess the benefits and EVLW and PVPI to assess the risks of pulmonary edema. Two randomized studies compared outcomes of critically ill patients monitored with pulmonary artery catheter to TPTD [89, 90]. Overall, no difference was found in outcomes including mortality, but in both studies the hemodynamic algorithms in the TPTD arm were of questionable value [91].

### Prediction of instability. Gleaning knowledge from data to predict CVI

Perhaps the newest frontier available to optimize care through precise and personalized monitoring is the use of machine learning approaches [92–96] to feature time series data to inform the bedside clinician of exactly the cardiovascular state of the patient and their most likely clinical trajectory. Predicting future hemodynamic events such as impending hypotension goes beyond the current practice of monitoring the current state of the patient. Untoward event prediction models are built on a training set using featurization of the specific hemodynamic monitoring data, either every few minutes, beat-to-beat or waveform and then tested on a separate validation set. Numerous groups have been using these approaches on large patient databases to show in silico their benefits when applied at the bedside by using retrospective data as their validation sets. Continuous vital sign data can create a fused vital sign index to predict CVI in step-down unit patients. When this smart bedside alert was coupled with a nursing action plan, the overall duration of CVI decreased 80% [97].

Different hypotension prediction models in various settings (perioperative, ICU) using different monitoring methods (invasive, non-invasive) to report a continuous hypotension prediction index (HPI) have been created. The best predictions of impending hypotension occur within 5–15 min of its occurrence, making them best suited for emergency and intra-operative care environments. One commercial HPI algorithm (Acumen™) uses both invasive and non-invasive estimates of the arterial pressure waveform [93]. Other models include a Super Learner or Supervised Machine-Learning Algorithm [92, 98], or Hybrid Deep Learning models [99] for detection of hypotension in the ICU [100]. Another three studies focused on the prediction of arterial hypotension occurring immediately after the induction of anesthesia (post-induction hypotension) [92, 101, 102]. The best predictive value (AUC 0.893) was found with the artificial neural network model developed by Lin et al. [102]. The Acumen HPI also predicted hypotensive episodes in cardiac surgery before and after cardiopulmonary bypass [103]. Retrospective analysis of large data demonstrated a strong relationship between the number and duration of hypotensive events (i.e., hypotensive burden) expressed as the time-weighted average (TWA) of a MAP beyond different thresholds and the number and duration of hypotensive. Most studies showed that using HPI, when coupled with a pre-emptive treatment reduced both the number of hypotensive episodes [104–106] and the TWA hypotension [104–106]. However, one study performed in 214 patients having moderate or high-risk noncardiac surgery failed to show benefit. Recent modifications of these measures using non-invasive monitoring inputs and in different OR and ICU populations are showing promising short-term results [105, 107–110].

## Conclusions

The proven efficacy of treatments based on specific hemodynamic monitoring have improved patient outcomes are small but relevant. Most studies identify improved process of care as surrogates for effectiveness, such as less volume of fluid infused and shorter time in hypotension. Importantly, the need for disease and process-specific clinical trials to include focused patient-centered outcome appears both warranted and essential if we are to continue to use monitoring to efficiently and effectively direct patient care and identify instability in the future. We are at the cusp of finding the optimal path where hemodynamic monitoring, coupled to an optimized patient care algorithm, produces the best clinical outcomes. A one-size-fits-all approach may prevent patients from receiving life-saving fluid and vasoactive medication administration when needed and may encourage the use of these interventions when futile.

## Data Availability

N/A, no new data were generated.
